# Single-photon absorption and emission from a natural photosynthetic complex

**DOI:** 10.1038/s41586-023-06121-5

**Published:** 2023-06-14

**Authors:** Quanwei Li, Kaydren Orcutt, Robert L. Cook, Javier Sabines-Chesterking, Ashley L. Tong, Gabriela S. Schlau-Cohen, Xiang Zhang, Graham R. Fleming, K. Birgitta Whaley

**Affiliations:** 1 Department of Chemistry, University of California, Berkeley, CA USA; 2 Kavli Energy Nanoscience Institute at Berkeley, Berkeley, CA USA; 3 Molecular Biophysics and Integrated Bioimaging Division, Lawrence Berkeley National Laboratory, Berkeley, CA USA; 4 Joint Quantum Institute, National Institute of Standards and Technology and University of Maryland, Gaithersburg, MD USA; 5 Department of Chemistry, Massachusetts Institute of Technology, Cambridge, MA USA; 6 Nanoscale Science and Engineering Center, University of California, Berkeley, CA USA

**Keywords:** Physical chemistry, Biophysics, Energy harvesting, Atomic and molecular physics

## Abstract

Photosynthesis is generally assumed to be initiated by a single photon^1–3^ from the Sun, which, as a weak light source, delivers at most a few tens of photons per nanometre squared per second within a chlorophyll absorption band^1^. Yet much experimental and theoretical work over the past 40 years has explored the events during photosynthesis subsequent to absorption of light from intense, ultrashort laser pulses^2–15^. Here, we use single photons to excite under ambient conditions the light-harvesting 2 (LH2) complex of the purple bacterium *Rhodobacter sphaeroides*, comprising B800 and B850 rings that contain 9 and 18 bacteriochlorophyll molecules, respectively. Excitation of the B800 ring leads to electronic energy transfer to the B850 ring in approximately 0.7 ps, followed by rapid B850-to-B850 energy transfer on an approximately 100-fs timescale and light emission at 850–875 nm (refs. ^16–19^). Using a heralded single-photon source^20,21^ along with coincidence counting, we establish time correlation functions for B800 excitation and B850 fluorescence emission and demonstrate that both events involve single photons. We also find that the probability distribution of the number of heralds per detected fluorescence photon supports the view that a single photon can upon absorption drive the subsequent energy transfer and fluorescence emission and hence, by extension, the primary charge separation of photosynthesis. An analytical stochastic model and a Monte Carlo numerical model capture the data, further confirming that absorption of single photons is correlated with emission of single photons in a natural light-harvesting complex.

## Abstract (web summary)

Using a heralded single-photon source along with coincidence counting, we establish time correlation functions for B800 excitation and B850 fluorescence emission and demonstrate that both events involve single photons.

## Main

**Fig. 1:**
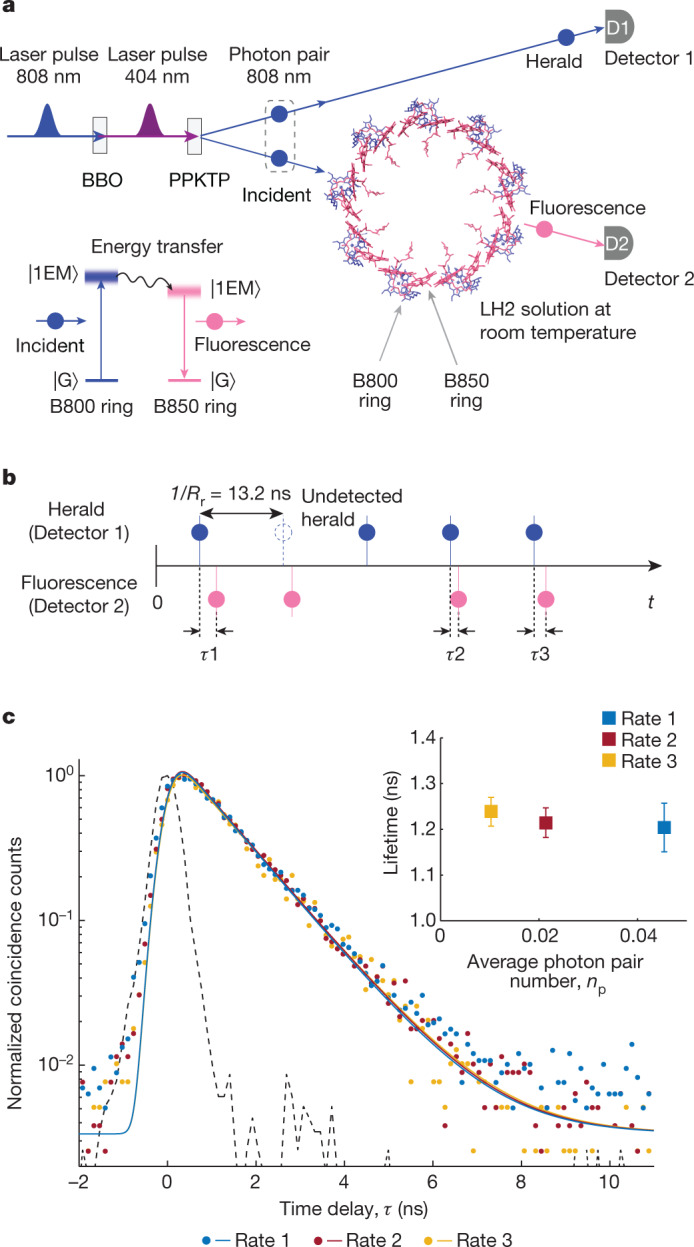
Principle of the experiments. **a**, Simplified schematic of the time-resolved PCQLS for studying single-photon transitions in photosynthetic systems. In the LH2 structure, the B800 ring (containing 9 bacteriochlorophylls) and the B850 ring (containing 18 bacteriochlorophylls) are colour coded as blue and red, respectively, produced from the Protein Data Bank file 1NKZ using ChimeraX. For simplicity, the carotenoids and protein subunits of LH2 are not shown here. The inset is a simplified energy diagram of LH2 showing the whole process from absorption of a single photon by the B800 ring to fluorescence of a single photon by the B850 ring after electronic energy transfer from B800. BBO, barium borate; PPKTP, periodically poled potassium titanyl phosphate; |G>, ground state; |1EM>, one-exciton manifold. **b**, Schematic of the raw signals showing the relative time delay *τ* between corresponding pairs of heralds and heralded fluorescent photons. **c**, Normalized coincidence counts of crosscorrelation between heralds and heralded fluorescent photons plotted as a function of their relative time delay *τ* with 128-ps bin size. The three sets of coloured dots show the measured data at three different incident photon rates represented by the average photon pair number *n*_p_ generated per pump pulse over a fourfold range (that is, 0.0458 (rate 1, blue), 0.0214 (rate 2, red) and 0.0103 (rate 3, orange), respectively, with 200-s integration time) (Supplementary Tables 2 and 3). The three solid curves are the corresponding single-exponential decay fits reconvoluted with the instrument response function (black dashed line) measured using the crosscorrelation between heralds and incident residue. The inset shows the obtained decay lifetimes, with error bars representing the 95% confidence intervals. Source data

Our measurements use time-resolved photon-counting quantum light spectroscopy (PCQLS) based on a heralded single-photon source^20,21^ and coincidence counting (Fig. 1, Supplementary Information Section I and Supplementary Fig. 1), with photon pairs around 808 nm produced from type II spontaneous parametric down-conversion in a nonlinear crystal pumped by a femtosecond laser with a repetition rate *R*_r_ = 75.7 MHz. The probability of having two simultaneously created pairs was maintained at a very small value (Supplementary Information Section II and Supplementary Fig. 2) as confirmed by measurements of the second-order coherence function at zero time delay, *g*^(2)^(*t* = 0), to be discussed later. One of the photons, the herald, was directly detected by a single-photon counting module, Detector 1, and signifies the presence of the other photon, which is focused by a home-built microscope into a solution of LH2 complexes under ambient conditions (Supplementary Fig. 3). After subsequent energy transfer and relaxation, a fluorescent photon emitted from the B850 ring was then collected, selected through spectral filters and detected by Detector 2. Finally, each of the detection events in each detector was time tagged with subnanosecond time resolution.

**Fig. 2:**
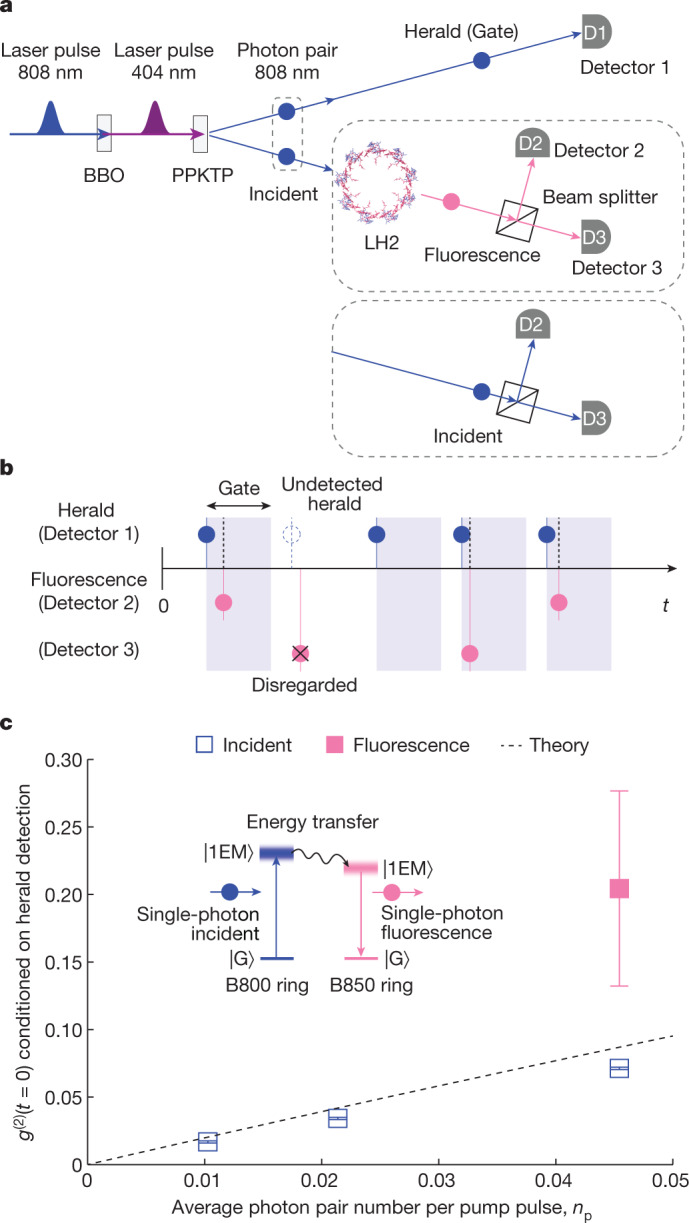
Single-photon nature revealed by the second-order coherence function at zero time delay, *g*^(2)^(*t* = 0), conditioned on herald detection. **a**, Schematics of the experiments to measure *g*^(2)^(*t* = 0) conditioned on herald detection by standard three-detector measurements. **b**, Schematics of the raw signal time trace. The gate window is 10 ns for fluorescence photons and 6 ns for incident photons. **c**, Measured values of *g*^(2)^(*t* = 0) conditioned on herald detection for incident and fluorescent photons. The conditional *g*^(2)^(*t* = 0) value of the heralded fluorescent photons, *g*^(2)^(*t* = 0) = 0.2044 ± 0.0723 (four s.d. below the single-photon threshold of 0.5), was calculated according to the equation *g*^(2)^(*t* = 0) = (*N*_H_ × *N*_C_)/(*N*_2_ × *N*_3_) (see refs. ^20,24^ for full details) using the following measured counts from a 5-h integration time: herald count *N*_H_ = 17,773,649,622, gated coincidence count between Detector 2 and Detector 3 *N*_C_ = 8, gated Detector 2 *N*_2_ = 819,108 and gated Detector 3 *N*_3_ = 849,299. The conditional *g*^(2)^(*t* = 0) values of heralded incident photons were measured for the same three rates as in Fig. 1c, each with 10-s integration time (Supplementary Information Section V and Supplementary Fig. 4). Error bars represent the s.d. assuming Poisson statistics of the counts. The black dashed line shows the theoretical estimate of *g*^(2)^(*t* = 0) = 2*n*_p_/(1 + *n*_p_) for heralded detections derived in Supplementary Information Section V. Source data

We first verified that the photons arriving at Detector 2 were indeed fluorescence signals by measuring their spectra (Supplementary Information Section III and Supplementary Fig. 3) and lifetime (Fig. 1b,c). As the signal from heralded single-photon excitation is very weak, the fluorescence spectra were measured under weak laser excitation for a better signal-to-noise ratio (Supplementary Fig. 3). The measured spectra showed a well-defined peak near 850 nm, characteristic of the fluorescence emission from the B850 ring in LH2 (refs. ^1,19^). After confirming the fluorescence spectra, we switched the excitation source to heralded single photons for PCQLS measurements correlating heralds of incident photons with fluorescent photons (Supplementary Information Section IV). At a photon pair production rate *R*_p_ = 3.47 × 10^6^ pairs per second as generated at the source (corresponding to an average photon pair number *n*_p_ = *R*_p_/*R*_r_ = 0.0458 generated per pump laser pulse) (Supplementary Fig. 2), we measured a herald rate *R*_h_ = 9.73 × 10^5^ counts per second at Detector 1. The measured rate of photons in the other channel that is incident on the LH2 sample, which we refer to as the incident rate, was *R*_i_ = 9.13 × 10^5^ counts per second (Supplementary Fig. 2). The small difference between the measured herald and incident rates is mainly due to the different collection efficiencies of the two optical modes. We detected a heralded fluorescent rate *R*_hf_ = 121 counts per second at Detector 2 within a 10-ns gate of herald detection after filtering out the residual incident photons (808 nm), yielding an overall heralded fluorescence channel efficiency *e*_hf_ = *R*_hf_/*R*_h_ = 1.244 × 10^−4^ that encapsulates all experimental inefficiencies from the photon pair source to Detector 2, including the internal electronic dynamics within the LH2 complexes. For all these measured count rates, the contributions from dark counts of the detectors were very small and can be safely ignored (Supplementary Table 2). Using the timing information of each detection event and the fact that the known fluorescence lifetime of LH2 (ref. ^19^) is much shorter than the laser repetition period 1/*R*_r_ = 13.2 ns, we then identified all corresponding pairs of herald and heralded fluorescent photons together with their relative time delay *τ* (Fig. 1b). This allowed us to construct the time-resolved second-order crosscorrelation between individual herald and heralded fluorescent photons. From this crosscorrelation, the fluorescent decay lifetime *τ*_0_ = 1.20 ± 0.05 ns was extracted using the single-exponential fluorescent intensity function *I*(*τ*) = *I*(0) × exp(−*τ*/*τ*_0_) after reconvolution with the instrument response function determined by the crosscorrelation between heralds and heralded incident residue (Fig. 1c). Additional crosscorrelations and lifetime data were acquired at two lower incident rates, allowing access to an overall fourfold range in incident rate. These data all showed a consistent single-exponential decay (Fig. 1c and Supplementary Table 3), with measured lifetime consistent with literature reports for fluorescence from B850 (ref. ^19^). Contributions to this fluorescence resulting from other states, such as long-lived triplets or charge transfer states, are excluded by quantum yield and timescale analysis, as has been discussed in the context of single-molecule studies of LH2 fluorescence^22^ (Supplementary Information Section IV).

We then verified the single-photon nature of the heralded incident and fluorescent photons by their second-order coherence function at zero time delay^23^, *g*^(2)^(*t* = 0), conditioned on herald detection using a standard three-detector measurement^20,24^ (Fig. 2). To this end, the fluorescent photons (and in a separate measurement, the incident photons (Supplementary Information Section V and Supplementary Fig. 4)) were split by a 50:50 beam splitter and detected by Detector 2 or Detector 3 on either side (Fig. 2a). A herald detection at Detector 1 provides a conditional gate for Detectors 2 and 3; only those photons arriving at Detectors 2 and 3 within the gate window (10 ns for fluorescence and 6 ns for incident) are counted (Fig. 2b). The conditional *g*^(2)^(*t* = 0) value, with ‘*t* = 0’ defined by the gate window, can then be obtained as^20,24^ *g*^(2)^(*t* = 0) = (*N*_H_ *×* *N*_C_)/(*N*_2_ *×* *N*_3_), where *N*_H_ is the herald count, *N*_C_ is the coincidence count between Detector 2 and Detector 3, and *N*_2_ (*N*_3_) is the count at Detector 2 (Detector 3). The measured conditional *g*^(2)^(*t* = 0) values of both heralded incident and heralded fluorescent photons are found to be much lower than the single-photon threshold of 0.5 (refs. ^20,24,25^), which unambiguously confirms both their single-photon nature and the very small probability of having two photons in either channel (Fig. 2c). We also derived a theoretical estimate for the heralded *g*^(2)^(*t* = 0) value, namely *g*^(2)^(*t* = 0) = 2*n*_p_/(1 + *n*_p_), following a simple model (described in Supplementary Information Section V), which agrees with the measured data (Fig. 2c).

The successful detection of heralded fluorescent single photons under heralded single-photon excitation in the PCQLS experiments provides strong evidence for both single-photon transitions in the initial light absorption process and a spatially separated single-photon absorption and emission cycle in photosynthetic systems. This is because at the pump laser repetition rate *R*_r_ = 75.7 MHz, the laser repetition period 1/*R*_r_ = 13.2 ns, which is the minimum time interval between successive incident single photons (the average time interval was greater than or equal to 288 ns given the photon pair production rate *R*_p_ ≤ 3.47 × 10^6^ pairs per second) (Supplementary Fig. 2e), was much longer than the fluorescent lifetime *τ*_0_ = 1.20 ± 0.05 of an LH2 complex. Any existing singlet excitation in the LH2 sample would thus have decayed before arrival of the next incident single photon. Consequently, in the absence of dark states (Supplementary Information Section IV), there can be essentially at most either one single photon or one single excitation in the entire LH2 ensemble at any given time, considering the very small probability of having more than one photon in each heralded incident photon pulse (Fig. 2c) and the fact that the sample contains on the order of 7 × 10^4^ LH2 complexes in the interaction volume (Supplementary Information Sections I and III). Therefore, observation of a heralded fluorescent single photon implies that one LH2 complex was previously in an excited state and that this excited state must have resulted from absorption of the entire energy of a heralded incident single photon in a single event (that is, via a single-photon transition).

**Fig. 3:**
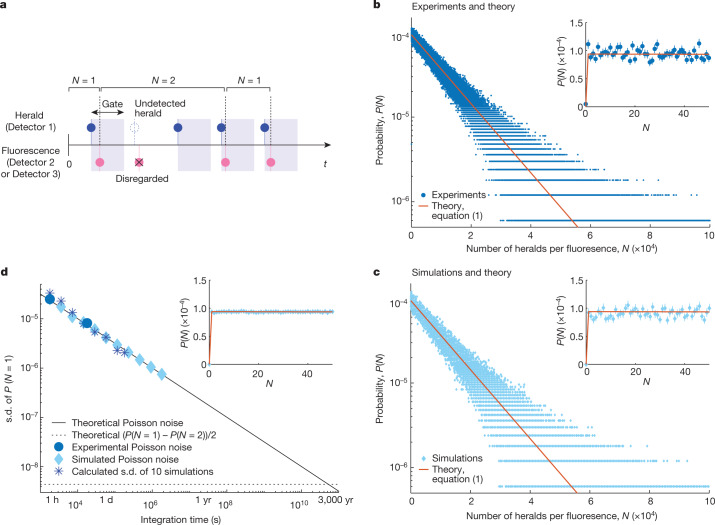
Probability distribution of the number of heralds per heralded fluorescent photon. **a**, Schematic of the raw signal time trace showing how the number of heralds between successive heralded fluorescent detections is counted. **b**,**c**, Experimental, theoretical and simulated probability distributions. **b**, The experimentally measured probability distributions from the same raw data used to extract the conditional *g*^(2)^(*t* = 0) of heralded fluorescent photons in Fig. 2c constructed from a total of 1,668,407 heralded fluorescent detections and a total of 17,773,649,622 herald detections, with 5-h integration time. Note here that the value *P*(*N* = 0) ≈ 4.8 × 10^−6^ is non-zero due to the eight events of two heralded fluorescent photons conditioned on a herald detection that also gave rise to the small non-zero *g*^(2)^(*t* = 0) value of the heralded fluorescence in Fig. 2c. The solid red curves show the prediction of the analytical stochastic model of equation (1) using the measured experimental parameters. **c**, Simulated probability distributions using a numerical Monte Carlo model (Supplementary Information Section VII) with experimental parameters from **b** shown together with the theoretical prediction of equation (1). The insets in **b** and **c** zoom into the first 50 bins, showing the data points together with error bars representing Poisson s.d. ($\sqrt{{\rm{c}}{\rm{o}}{\rm{u}}{\rm{n}}{\rm{t}}{\rm{s}}\,{\rm{i}}{\rm{n}}\,{\rm{b}}{\rm{i}}{\rm{n}}\,N}$ normalized by the total counts of all bins). **d**, The s.d. of *P*(*N* = 1) as a function of the integration time showing that the Poisson counting noise (defined as $\sqrt{{\rm{c}}{\rm{o}}{\rm{u}}{\rm{n}}{\rm{t}}{\rm{s}}\,{\rm{i}}{\rm{n}}\,{\rm{b}}{\rm{i}}{\rm{n}}\,N=1}$ normalized by the total counts of all bins) can be reduced by longer integration time and thus, more total events. The inset zooms into a simulated distribution with 1.8 × 10^6^-s integration time (100 times longer than the experimental and simulated data in **b** and **c**) and shows much reduced noise (Supplementary Information Fig. 8). Error bars represent Poisson s.d. Source data

To further quantitatively validate this conclusion of single-photon transitions, we constructed the probability distribution of the number of heralds between successive heralded fluorescent single photons from the time-tagged data (Fig. 3a, Supplementary Information Section VI and Supplementary Fig. 5). Such a distribution constructed from a total of 1,668,407 detected heralded fluorescent events and a total of 17,773,649,622 detected heralds is shown in Fig. 3b, from which it is evident that the maximum probability occurs at close to one herald. This is additional strong evidence for single-photon transitions initiating photosynthetic energy transfer and a single-photon absorption and spatially separated single-photon emission cycle. We note that the maximum probability in an experimental distribution may not be at exactly one herald due to the Poisson counting noise. This is particularly pronounced at small *e*_hf_ values, which will be discussed in more detail below (Fig. 3c,d).

We also studied the dependence of the probability distribution on the probability weight of multi-photon components in the heralded incident single photons by lowering the rate of incident photons by up to a factor of four (Supplementary Fig. 6 and Supplementary Table 4). While the distribution was somewhat noisier at lower incident rates, consistent with a smaller total number of detection events, the shape remained unchanged. This is consistent with the fact that the heralded fluorescent channel efficiency *e*_hf_ was essentially constant (Supplementary Table 2). Importantly, it rules out any possible nonlinear or multi-photon contributions being the main cause of the observed fluorescent photons under these experimental conditions. In particular, any contribution from the tiny multiple-photon components in the heralded incident single photons can be excluded. This is because the probability weight of the *M*-photon components scale as *n*_p_^*M−*1^/(1 + *n*_p_)^*M*^, (*M* = 1, 2, 3 …), with *n*_p_ = *R*_p_/*R*_r_ (less than or equal to 0.0458 in our experiments) being the average number of photon pairs produced by each pump laser pulse, resulting from the fact that the generated photon pair can be described by a squeezed vacuum state^25,26^. A detailed analysis of the relationship of *e*_hf_ to *n*_p_ is given in Supplementary Information Section IV.

The measured distribution in Fig. 3b can be quantitatively explained by an analytical stochastic model. This establishes a geometric distribution of the herald number with heralded fluorescent photon probability *e*_hf_. The probability, *P*(*N*), of the distribution at the number, *N*, of heralds per each heralded fluorescent photon can then be shown to be (Supplementary Information Section VI)

1a $$P(N)={e}_{{\rm{h}}{\rm{f}}}{(1-{e}_{{\rm{h}}{\rm{f}}})}^{N-1},\,{\rm{f}}{\rm{o}}{\rm{r}}\,N=1,\,2,\,3\ldots ,$$

1b $$P\left(N=0\right)=0.$$

The most important feature of equation (1) is that the maximum probability occurs at *P*(*N* = 1) for *N* ≥ 1, as expected from a single-photon fluorescence subsequent to a single-photon absorption. A second key feature is that for very small heralded fluorescence efficiencies *e*_hf_ << 1, the geometric distribution in equation (1a) reduces to a single-exponential decay with *P*(*N*) ∝ exp(−*Ne*_hf_), which agrees well with fits of the experimental distributions (Supplementary Fig. 6 and Supplementary Table 4). Note that the experimental probability in the zeroth bin, *P*(*N* = 0), is not exactly equal to zero due to the small finite probability of two fluorescent photons conditioned on the same herald, included in our counting scheme (Fig. 3a and Supplementary Information Section VI). These are the same events that give rise to the small non-zero *g*^(2)^(*t* = 0) value of the heralded fluorescence in Fig. 2c. In Fig. 3b, we plot the analytical *P*(*N*) from equation (1) evaluated with the measured *e*_hf_. The plots show excellent agreement between the experiments and the analytical stochastic model, which further supports our experimental inference of single-photon transitions in absorption and a single-photon absorption and emission cycle.

In addition to the analytical model, we also carried out numerical Monte Carlo calculations to simulate the experimental results with individual stochastic trajectories using the experimental parameters (Supplementary Information Section VII). As shown in Fig. 3c, the numerical simulation is in excellent agreement with the analytic stochastic model. We note that the simulated distribution displays the same level of Poisson counting noise as the experimental distribution in Fig. 3b. Performing additional simulations with significantly longer integration time and thus, significantly more total detection events than the experiments, we show that the Poisson counting noise can be reduced (Fig. 3d and Supplementary Information Fig. 8). Overall, Fig. 3 shows that the experimental results, the analytical model from equation (1) and the numerically simulated probability distribution all agree very well with each other, validating the experimental observations as reflecting single-photon absorption by the B800 ring correlated with subsequent single-photon fluorescence by the B850 ring.

We emphasize that this PCQLS method is distinct from the conventional time-correlated single photon counting measurements, which use classical laser pulses to excite the sample and thus, cannot provide information on the correlations between individual incident and fluorescent single photons. By allowing us to time, count and correlate individual incident photons and individual fluorescent photons, PCQLS has made it possible to directly show on an ensemble of LH2 pigment–protein complexes from purple bacteria *Rhodobacter sphaeroides* under ambient conditions that photosynthesis is initiated by absorption of a single photon and then proceeds on the basis of the associated single quantum of energy. Our results are particularly relevant for in vivo conditions because natural sunlight has a very low flux, about 60 photons per nm^2^ per second for a 20-nm bandwidth at 808 nm at the full power of the sun^1^, similar to the single-photon flux used in our experiments.

The PCQLS approach should enable new experiments capable of tracking single-excitation pathways in photosynthesis and other photo-driven processes in complex systems. Extending PCQLS to a pump–probe setup and applying it to photosynthetic complexes could reveal the excitonic dynamics after initial absorption, with femtosecond-timing resolution and/or spatial–temporal information on the interplay between initial light absorption and subsequent energy transfer. Spectrally resolving the fluorescent photons from complexes with clearly defined energy funnels^27^ could in principle allow greater spatial resolution of the pathways followed by individual excitations. We anticipate further advances from exploiting the new spectroscopic variables available from quantum light-based studies^28,29^. For example, entanglement in the photon pair could be exploited to improve spectral and temporal resolution and to break the classical Fourier transform limit^30,31^. Comparing photosynthetic light harvesting driven with laser, single-photon^32^ and thermal light^33^, respectively, could clarify the differences between in vitro and in vivo studies. Measuring the photon statistics of frequency-resolved fluorescence could provide new insights into the role of quantum coherence in excitonic energy transfer^34,35^. In short, we anticipate that similar to the way in which single-molecule experiments opened new avenues of investigation, PCQLS will prove a valuable and unique tool for exploring the spatio–temporal dynamics of individual excitations in a broad range of complex biological, chemical and physical systems and thereby gaining insight into their behaviour.

## Online content

Any methods, additional references, Nature Portfolio reporting summaries, source data, extended data, supplementary information, acknowledgements, peer review information; details of author contributions and competing interests; and statements of data and code availability are available at 10.1038/s41586-023-06121-5.

## Supplementary information

Supplementary Information

Supplementary Code 1 The script HFProbDist_Rept.m calls the function HFProbDist.m multiple times and writes data into files.

Supplementary Code 2 The function HFProbDist.m uses a numerical Monte Carlo model to simulate the probability distributions of the number of heralds per heralded fluorescent photon.

## Data Availability

The authors declare that the data supporting the findings of this study are available in the article and its supplementary information files. Source data are provided with this paper.

## Source data

Source Data Fig. 1
Source Data Fig. 2
Source Data Fig. 3

## Code availability

The custom code for the numerical Monte Carlo simulations that support the findings of this study is provided with this paper.
