# Supplementary material for: Single-photon absorption and emission from a natural photosynthetic complex
Source: Nature. 2023 Jun 14;619(7969):300–4. doi: 10.1038/s41586-023-06121-5 (PMC10338339; doi:10.1038/s41586-023-06121-5)
Supplement: Supplementary file 1 — Supplementary Information [file 41586_2023_6121_MOESM1_ESM.pdf]

nature portfolio

https://doi.org/10.1038/s41586-023-06121-5

Supplementary information

Single-photon absorption and emission from a natural photosynthetic complex

In the format provided by the authors and unedited

Supplementary Information for: "Single-photon absorption and emission from a natural photosynthetic complex"

Quanwei Li,[1,2] Kaydren Orcutt,[1,2,3] Robert L. Cook,[1,2] Javier Sabines-Chesterking,[4] Ashley L. Tong,[5] Gabriela S. Schlau-Cohen,[5] Xiang Zhang,[2,6] Graham R. Fleming,[1,2,3] and K. Birgitta Whaley[1,2]

[1]*Department of Chemistry, University of California, Berkeley, CA 94720, USA*
[2]*Kavli Energy Nanoscience Institute at Berkeley, Berkeley, CA 94720, USA*
[3]*Molecular Biophysics and Integrated Bioimaging Division, Lawrence Berkeley National Laboratory, Berkeley, CA 94720, USA*
[4]*Joint Quantum Institute, National Institute of Standards and Technology and University of Maryland, Gaithersburg, MD 20899, USA*
[5]*Department of Chemistry, Massachusetts Institute of Technology, Cambridge, MA 02139, USA*
[6]*Nanoscale Science and Engineering Center, University of California, Berkeley, CA 94720, USA*

(Dated: March 15, 2023)

CONTENTS

I. The experimental setup 2
A. Summary table of definitions 4

II. Characterization of the photon pair source 5

III. The LH2 sample: preparation, structure and fluorescence spectrum 7

IV. Heralded fluorescent photons from heralded single-photon excitation 8

V. The second order coherence function at zero time delay $g^{(2)}(t=0)$ of the heralded incident photons and the heralded fluorescent photons 10
A. Measured values of $g^{(2)}(t=0)$ of heralded incident photons 10
B. Measured value of $g^{(2)}(t=0)$ of heralded fluorescent photons 10
C. Theoretical estimation of $g^{(2)}(t=0)$ 11

VI. Derivation of probability distribution of the number of heralds per fluorescent photon 12
A. Counting Scheme 12
B. Derivation of Eq. (1) in the main text and its asymptotic form 12
C. Theoretical estimation of $P(0)$ considering multi-photon events in the heralded incident photons 13

VII. Numerical simulations of the probability distribution of the number of heralds per fluorescent photon 14
A. Numerical simulation methodology 14
B. Comparison of experimental distributions with simulated distributions for same data integration times 14
C. Distribution of the simulated counts in the $N=1$ bin 16
D. Longer time numerical simulations of the probability distribution of the number of heralds per fluorescent photon 17

References 18

I. THE EXPERIMENTAL SETUP

The photon-counting quantum light spectroscopy (PCQLS) setup is built to be high-performance, modularized, and versatile (Fig. S1). The setup consists of three major optical modules – an SHG (second harmonic generation) module to prepare the pump laser, an SPDC (spontaneous parametric down conversion) module to generate the photon pairs, and a fluorescent module for fluorescence excitation and collection – and the detection electronics.

A fs laser with repetition rate $R_r = 75.7$ MHz (Mira 900, Coherent) at 808 nm is first converted up to 404 nm via a barium borate (BBO) crystal in the SHG module. After spatially removing the 808 nm laser residue by a prism and further blocking it by a spectral filter (FF01-424/SP-25, Semrock), the 404 nm laser is then coupled into single-mode fiber (SMF) and directed to the SPDC module to produce photon pairs at 808 nm in a 30 mm long periodically poled potassium titanyl phosphate (PPKTP) crystal (poling period 9.825 μm) (Raicol Crystals Ltd) [1]. Additional filters (FF01-424/SP-25 and FF01-400/40-25, Semrock) are used before the 404 nm laser is incident onto the PPKTP crystal. A set of five filters (LP610-25.4, MIDOPT; BLP02-561R-25, BLP01-647R-25, BLP01-473R-25 and FF01-842/SP-25, Semrock) are used after the PPKTP crystal to filter out the residual 404 nm laser while transmit the generated photon pair. In the collinear type II SPDC process, the signal and idler photons have orthogonal polarizations and are spatially separated deterministically by a polarizing beam splitter (PBS) and collected into SMFs. One of the two photons is detected as the herald by single-photon counting module Detector 1 (SPCM AQRH 13, Excelitas). The other single photon is directed to the fluorescence module.

In the fluorescence module, the 808 nm photon is reflected by a dichroic mirror (Di02-R830-25x36, Semrock), coupled into a microscope objective (60×, NA = 0.70) and finally incident on the LH2 sample. The sample contains $\sim 7 \times 10^4$ LH2 complexes in solution in the interaction volume, estimated from the concentration 31.4 μM with a volume of $\sim 3.6\mu m^3$ that interacts with the light. Details of the sample preparation and characterization are provided in Section III. The LH2 solution sample is sandwiched between a SiO_2 coated silver mirror (PF10-03-P01, Thorlabs) and a cover glass (Fig. S3 **c**), and stays at room temperature under ambient conditions. The sample and mirror are on a manual 3-axis translation stage. Both fluorescence and residual incident 808 nm photons are collected by the same objective and transmit through the dichroic mirror. Then, either fluorescent photons or residual incident photons are spectrally selected by a proper filter (LP02-830RU-25 or FF01-810/10-25, Semrock, respectively) that blocks the other. Those photons are collected by a 50-um core multi-mode fiber (MMF) connected to single-photon counting module Detector 2 (SPCM AQRH 16, Excelitas). Each detection event from the two detectors is recorded with ~ 350 ps resolution by a time tagger (Time Tagger Ultra, Performance Edition, Swabian Instruments) with channel dead time setting $T_D = 86$ ns.

For measuring the second order coherence function at zero time delay $g^{(2)}(t = 0)$ of the fluorescent photons conditioned on herald detection, the fluorescent photons are split by a 50:50 fiber beam splitter and the two output ports are connected to single-photon counting modules Detector 2 (SPCM AQRH 16, Excelitas) and Detector 3 (SPCM AQRH 16, Excelitas), respectively. As above, each detection event from the three detectors is recorded with ~ 350 ps resolution by a time tagger (Time Tagger Ultra, Performance Edition, Swabian Instruments) having channel dead time setting $T_D = 86$ ns.

Fig. S1 shows the three major optical modules of the PCQLS setup used in this work. The bottom right panel (yellow) shows the SHG module. The top panel (cyan) shows the SPDC module. The bottom left panel (magenta) shows the fluorescence module. The curved arrows show the optical pathway connections that are used for the configuration of PCQLS to study single-photon quantum transitions. These connections are made with optical fibers (SMF, yellow; MMF, orange). The optics inside the dotted box and the flip mirror in the fluorescence module are used for alignment inspection. The optics inside the dashed box (including Fiber BS and D3) are plugged into the setup when measuring the second order coherence function at zero time delay $g^{(2)}(t = 0)$ of the fluorescent photons. Filters1: FF01-424/SP-25, Semrock. Filters2: FF01-810/10-25, Semrock. Filters3: FF01-424/SP-25 and FF01-400/40-25, Semrock. Filters4: LP610-25.4, MIDOPT; BLP02-561R-25, BLP01-647R-25, BLP01-473R-25 and FF01-842/SP-25, Semrock. Filters5: LP02-830RU-25 (for selecting fluorescence) or FF01-810/10-25 (for selecting incident residue), Semrock. Dichroic mirror: Di02-R830-25x36, Semrock. We use the following abbreviations: HWP = half-wave plate, PBS = polarizing beam splitter, BBO = barium borate (BaB2O4), SMF = single-mode fiber, PPKTP = periodically poled potassium titanyl phosphate, MMF = multimode fiber, BS = beam splitter.

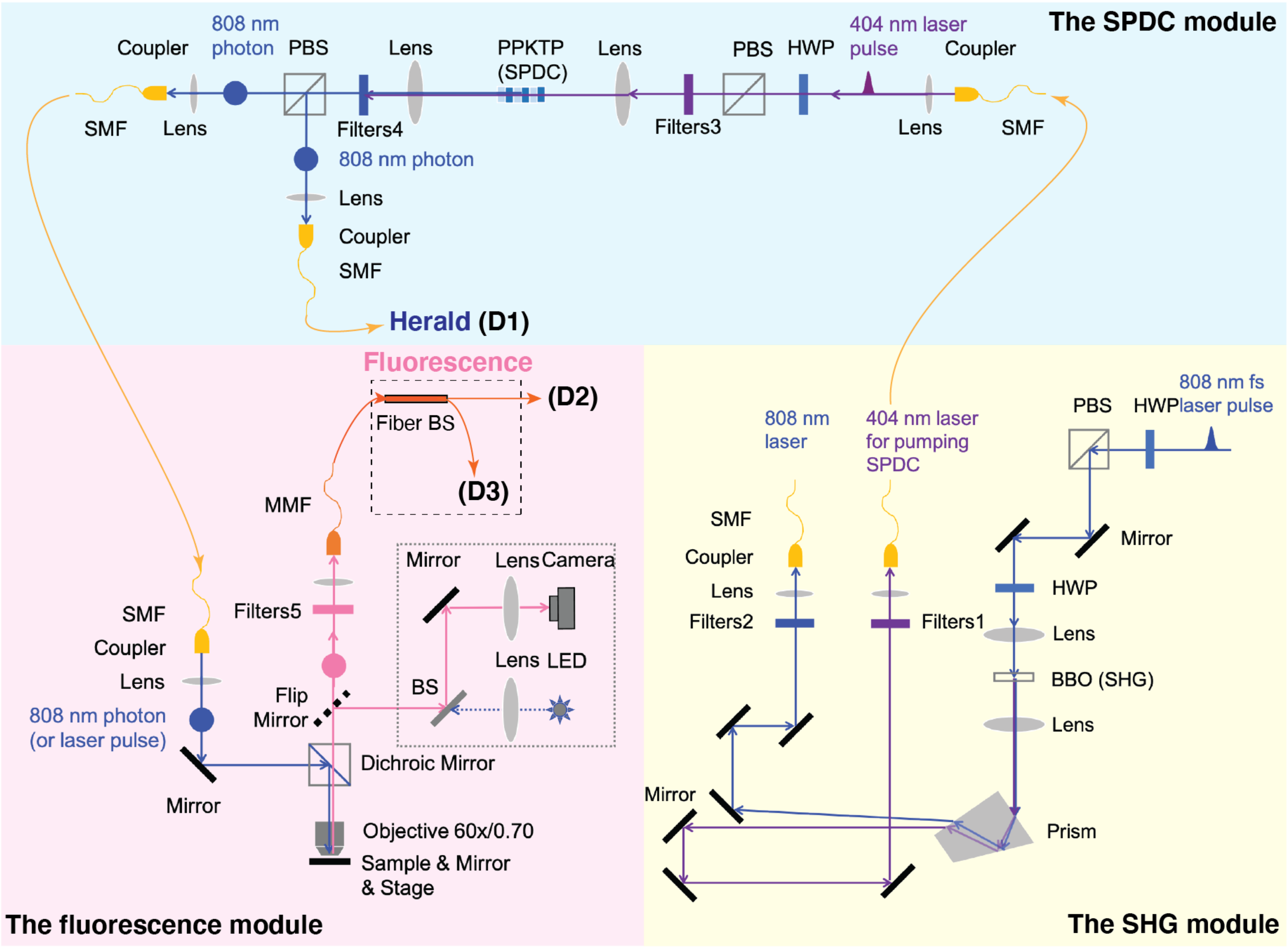

Supplementary Figure S1: **Schematic layout of the major optical modules of the photon-counting quantum light spectroscopy (PCQLS) setup.**

A. Summary table of definitions

Experimental parameter	Symbol	Relation with other parameters	Value
Measured pump laser repetition rate (MHz)	R_r	–	75.7
Measured herald (idler) photon rate (counts/s)	R_h	–	see Fig. S2
Measured incident (signal) photon rate (counts/s)	R_i	–	see Fig. S2
Measured photon pair coincidence rate (pairs/s)	R_c	–	see Fig. S2
Calculated photon pair production rate as generated at the PPKTP crystal (pairs/s)	R_p	$R_p = R_h \times R_i/R_c$	see Fig. S2
Calculated average number of photon pairs produced per 404 nm laser pump pulse	n_p	$n_p = R_p/R_r$	see Fig. S2
Calculated herald (idler) channel efficiency	e_h	$e_h = R_h/R_p = R_c/R_i$	see Fig. S2
Calculated incident (signal) channel efficiency	e_i	$e_i = R_i/R_p = R_c/R_h$	see Fig. S2
Measured heralded fluorescent rate (counts/s)	R_{hf}	–	see Table S2
Calculated efficiency of heralded fluorescence	e_{hf}	$e_{hf} = R_{hf}/R_h$	see Table S2

Supplementary Table S1: Summary table of definitions of the main experimental parameters used in the main text and supplementary information.

II. CHARACTERIZATION OF THE PHOTON PAIR SOURCE

Fig. S2 summarizes the experimental procedures used to characterize the photon pair source and the results obtained for the three incident photon rates used in the experiments. The 404 nm laser pump power determines the average rate of production of photon pairs, R_p, at the PPKTP crystal and of measured herald, R_h, measured incident photons, R_i, and measured coincidence rate, R_c, between herald and incident photons. We refer to experiments under different 404 nm pump powers as experiments under different average rate of incident photons.

Panel **a** shows a schematic layout of the photon pair source setup (upper section, cyan) and detection electronics (lower section, blue). Photon pairs at 808 nm wavelength are generated from type II SPDC (spontaneous parametric down conversion) in a PPKTP (periodically poled potassium titanyl phosphate) crystal (Section I). Filters3: FF01-424/SP-25 and FF01-400/40-25, Semrock. Filters4: LP610-25.4, MIDOPT; BLP02-561R-25, BLP01-647R-25, BLP01-473R-25 and FF01-842/SP-25, Semrock. We use the abbreviations SMF = single-mode fiber, HWP = half-wave plate, PBS = polarizing beam splitter.

Panel **b** shows a typical measurement of coincidence counts between the herald and incident photons (i.e. between idle photon at Detector 1 and signal photon at Detector 2) as a function of their relative time delay. The main peak at zero-time delay correlates two photons emitted from the same pair, while the smaller side peaks spaced with $1/R_r =$ 13.2 ns intervals come from the periodic time-shifted random coincidence between photons from different pairs. The grey-shadowed region (6 ns) marks the time window used for calculating the coincidence count rates in panel **c**. In the PCQLS experiments the idler photons are used as herald photons and the signal photons are incident on the sample and referred as incident photons.

Panel **c** shows the measured count rates as a function of the power of the 404 nm laser pump (top axis) and calculated average photon pair number, $n_p = R_p/R_r$, generated per pump pulse (bottom axis) for i) measured herald photons, R_h, ii) measured incident photons, R_i, iii) measured 2-photon coincidence of photons deriving from the photon pair source after being collected into single mode fibers, R_c, and iv) calculated photon pair generation count rates at the PPKTP crystal, $R_p = R_h \times R_i/R_c$.

Panel **d** shows the calculated efficiencies of the herald and incident photons, defined as the herald (incident) rate divided by the calculated photon pair generation rate, i.e., $e_h = R_h/R_p$ and $e_i = R_i/R_p$.

Panel **e** shows the calculated average time interval between successive photons (pair) for herald ($1/R_h$), incident ($1/R_i$), and pair generation ($1/R_p$).

The small difference between the measured herald and incident rates (panel **c**) is due to the slightly different collection efficiencies of the two optical modes. The probability of having two simultaneously created pairs was kept at a very small value by ensuring that the average number of photon pairs produced per 404 nm laser pump pulse is much less that one, i.e., $n_p \ll 1$, and was also directly confirmed by measuring the second order coherence function of the incident photons at zero time delay conditioned on herald detections. The latter values are presented in Fig. 2c in the main text and in Section V below.

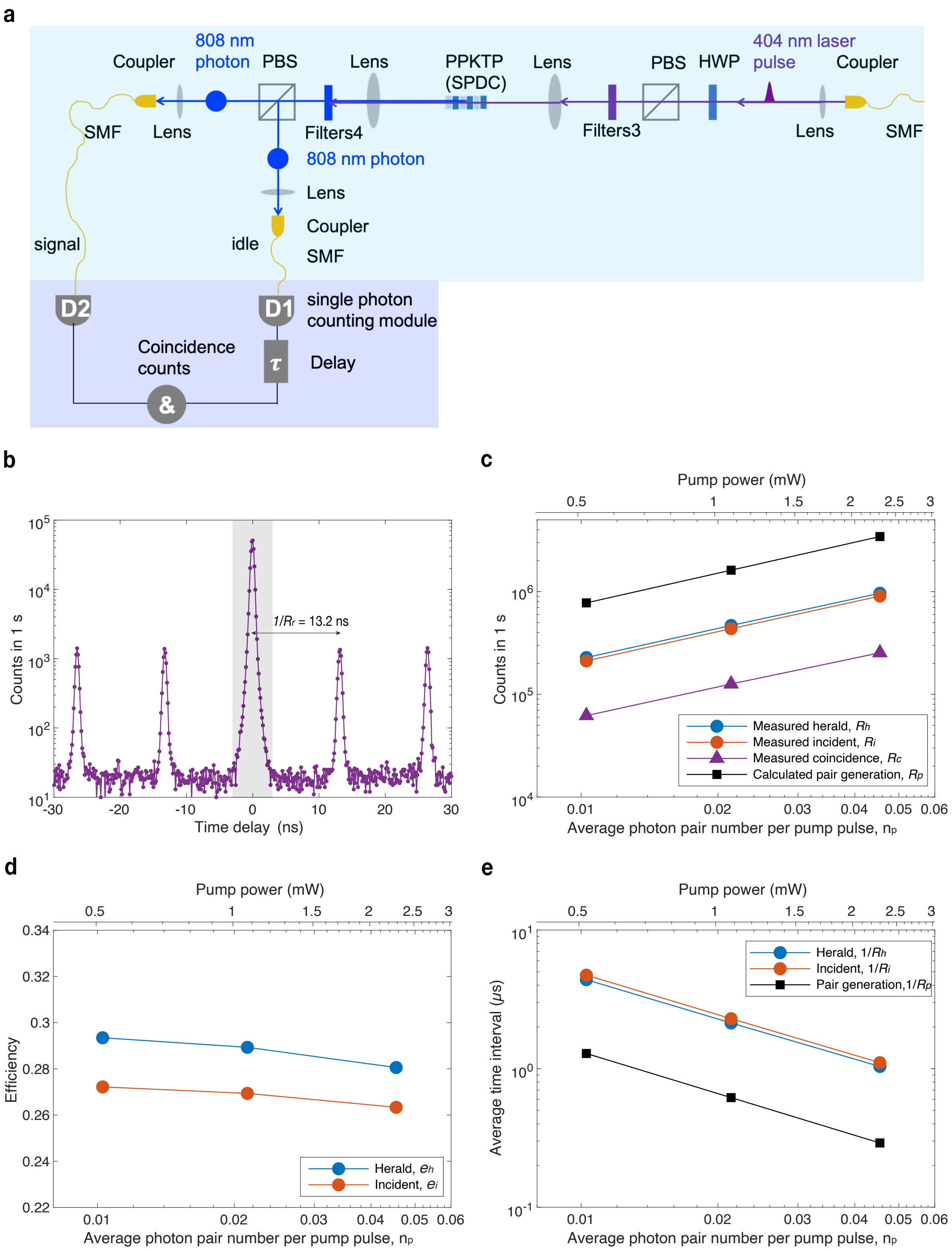

Supplementary Figure S2: Characterization of the photon pair source.

III. THE LH2 SAMPLE: PREPARATION, STRUCTURE AND FLUORESCENCE SPECTRUM

LH2 was purified as previously described from wild-type *Rhodobacter (R.) sphaeroides* ATCC2.4.1 cells provided by the Blankenship Lab at Washington University in St. Louis [2,3]. Purified LH2 complexes were solubilized in 20 mM Tris HCl, 0.1% DDM, pH 7.5 buffer, concentrated to 31.4 μM, and stored at -80°C in 500-μL aliquots until used for experiments.

Fig. S3 summarizes the structure of the LH2 complex and shows a zoom-in of the experimental schematic to excite and collect the fluorescence, together with the measured fluorescence spectrum of the sample.

Panel **a** shows the molecular structure of a single LH2 complex from two different perspectives, perpendicular and parallel to the two component rings of chlorophyll molecules. The B800 ring contains 9 bacteriochlorophyll molecules (blue) and absorbs at approximately 800 nm. The B850 ring contains 18 bacteriochlorophyll molecules (red) and absorbs at approximately 850 nm [2,3,4]. For simplicity, the carotenoids and protein subunits of LH2 are not shown here. The LH2 structure is produced from the Protein Data Bank file 1NKZ using ChimeraX.

Panel **b** shows an energy diagram of LH2 indicating absorption by the B800 ring and subsequent fluorescence by the B850 ring after ultrafast energy transfer from B800. Here |G> denotes the ground state and |1EM> the one-exciton manifold.

Panel **c** shows a zoom-in schematic of the fluorescence excitation and collection setup. We employ a high numerical aperture objective and carefully selected optics that optimize the collection efficiency, stability, as well as convenience of operation and system integration.

Panel **d** shows the fluorescence spectrum of the LH2 ensemble sample under ambient conditions *in vitro*. For the fluorescence spectral measurements a weak laser source at 808 nm (the "808 nm laser" port in the SHG module, Fig. S1) was used to excite the LH2 sample in order to obtain a better signal-to-noise ratio. Any residual incident laser light at 808 nm (incident residue) is also reflected and then can be filtered out when measuring the fluorescence (Section I). The red (blue) curve shows the fluorescence spectra under laser excitation with (without) filtering out the residual laser light at 808 nm. This is the raw data taken by a spectrometer (USB4000 Fiber Optic Spectrometer, Ocean Optics) with its native electric dark correction option enabled and no normalization or any other postprocessing of the data. The data can extend to negative values due to the factory setting and/or the specific algorithm of the spectrometer. The characteristic peak around 850 nm [2,3,4] in panel **d** confirms that the fluorescence emission is indeed from the B850 ring.

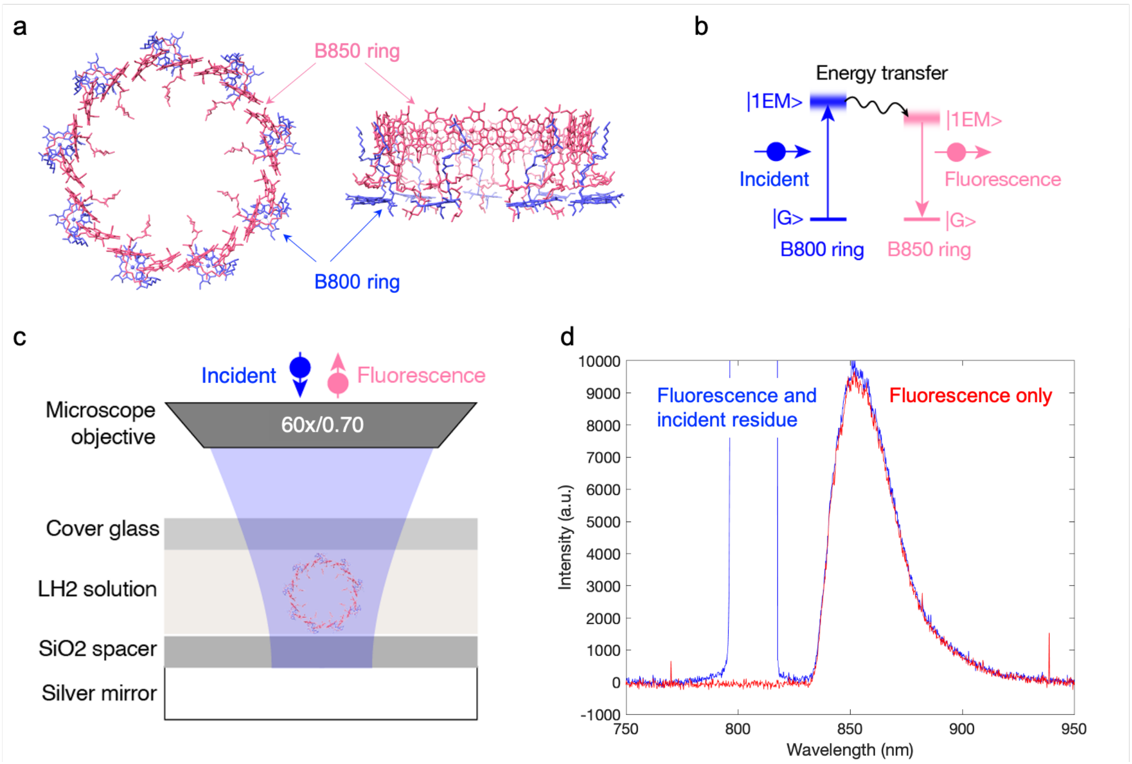

Supplementary Figure S3: Structure and fluorescence spectrum of the LH2 sample.

IV. HERALDED FLUORESCENT PHOTONS FROM HERALDED SINGLE-PHOTON EXCITATION

Table S2 lists the operating parameters for measurements of the heralded fluorescent photons from the LH2 sample under heralded single-photon excitation. The heralded fluorescence efficiency e_{hf} is defined in terms of the average number of fluorescent photons detected within a 10 ns gate window following each herald detection, i.e., $e_{hf} = R_{hf}/R_h$. This can be broken down into the product of the probability of absorption of a single photon by the sample , p_{ab}, with the probability of subsequent emission of a fluorescent photon, and the combined efficiency of the photon collection and detection system. The product of the latter two terms equal to the product of the heralding efficiency of the incident photons, e_i, with the fluorescent quantum yield of LH2 for emission from the B850 ring, and the overall transmission of the optical set up, which includes the incident photon transmission, fluorescence transmission and fluorescence collection. We estimate an approximate value of $p_{ab} \sim 0.5$ for the fraction of the sample that interacts with the light, using the literature value of $\sim 10^{-6}$ for probability of absorption by a single chlorophyll molecule [5] and scaling this up to the total number of B800 chlorophylls in the sample volume interacting with the incident single photons. This contains $\sim 7 \times 10^4$ LH2 complexes in solution, estimated from the concentration 31.4 μM with a volume of $\sim 3.6 \mu m^3$ that interacts with the light. The product of $p_{ab} \sim 0.5$, with the heralding efficiency of the incident photons $e_i \sim 0.26$, and the known fluorescent quantum yield ~ 0.2 of LH2 for emission from B850 chlorophylls [6] with the estimated transmission of the optic setup ~ 0.01 (including incident photon transmission, fluorescence collection and transmission) leads to an estimated fluorescence efficiency $e_{hf} \sim 2.6 \times 10^{-4}$. This value is in good agreement (i.e., with a factor ~ 2) with the value of $e_{hf} \sim 1.2 \times 10^{-4}$ that is obtained from the ratio of measured heralded fluorescent photon count rate to herald count rate, R_{hf}/R_h, shown in Table S2 below.

The detector dark counts follow Poisson distributions and the measured dark count rates in Table S2 are long time averages of these. Because of the gated nature of the heralded fluorescent photon detection, the contribution from dark counts is found to be only $< 0.3\%$ of the total counts and thus can be safely ignored.

We note that the obtained e_{hf} values appear to show a small (i.e., few percent) increase as the incident rate increases. This weak trend may be explained by analysis of the average photon number, $\bar{n}$, in the heralded incident single-photon pulse. Following a simple model described in Section V.C below, in particular making use of Eq. (4), we see that $\bar{n} = 1 + n_p$, where n_p is the average number of photon pairs per pulse (Sec. II). For linear absorption, it then follows that $R_{hf} \propto (1 + n_p)$ and hence also that $e_{hf} \propto (1 + n_p)$. In this sense, the $\sim 3.3\%$ increase of e_{hf} seen in Table S2 from Rate 3 to Rate 1 is consistent with the change of n_p from ~ 0.01 for Rate 3, to ~ 0.046 for Rate 1. We emphasize that, this weak dependence of e_{hf} on the value of n_p derives from the projection of photon number in the incident beam and does not imply any non-linear contribution. For a non-linear contribution due to $n > 1$ photons, the e_{hf} values would increase according to the corresponding multi-photon probability in the heralded incident pulse, $P_h(n_p, n)$, which follows a power law in n (see Eq. (3) in Sec. V.C). Any non-linear contribution from $n > 1$ would result in a significant increase of e_{hf} over the 4-fold range of n_p values presented in Table S2 and Fig. S2. For example, a non-linear contribution from $n = 2$ would give about 4.5-fold increase in e_{hf}, rather than a 3.3% increase, while a non-linear contribution from $n = 3$ would give about 16-fold increase.

We also note that while there are two potential long-lived states in LH2 of *R. sphaeroides*, namely triplet states of bacteriochlorophyll (^{3}BChl) or of the carotenoid sphaeroidene (^{3}Car), and BChl anions resulting from charge transfer between BChl and Car [7], none of these long-lived states can contribute to the observed fluorescence in this experiment. BChl anions can be ruled out immediately since the timescale for recombination of the charge transfer states, leading to ground state BChl and Car, is 8 ps for BChl/CAR [8]. The triplet states ^{3}BChl and ^{3}Car both have similar energy to that of the BChl Qy state, so that if a second 800 nm photon arrives, this could either excite the BChl Qy state to a higher lying singlet state or excite one of the triplet states of BChl or Car to a higher lying triplet. BChl Qy could then be repopulated by intersystem crossing to a higher singlet state from such a higher lying triplet, followed by relaxation or emission to BChl Qy, thereby enabling additional energy transfer to B850 and additional fluorescence emission of this. However, such scenarios are exceedingly unlikely in our experiment since the quantum yield of triplet formation is very low, of order 2% [9]. Consequently, with $\sim 7 \times 10^4$ LH2 molecules in our focal volume, the probability of a second photon interacting with a previously excited LH2 that has formed a ^{3}BChl triplet state is of order 10^{-7}, even before taking into account the average time between incident photons ($\simeq 1\mu$s, see panel c of Fig. S2) and the triplet lifetime of $\sim 2 - 8\mu$s [9]. We are therefore confident that we can rule out the presence of long-lived dark states contributing to our fluorescence detection events.

Table S3 lists the experimental parameters and data of the measurements of the time-resolved second order cross correlation between individual herald and heralded fluorescent photons used to generate the fluorescence lifetime plots in Fig. 1c of the main text. The bin size was 128 ps. The instrument response function was measured for the highest incident rate (Rate 1) only. We measured a heralded incident residual rate of approximately 60 counts/s at Detector 2 within 6 ns gate of herald detection by using a different filter through which only the 808 nm incident photons can pass (see Section I). The signals from heralded incident residual photons are transmitted through the entire setup and detection electronics. Thus, they can serve as the instrument response function for lifetime measurements.

Incident rates	Rate 1	Rate 2	Rate 3
Calculated average number of photon pairs produced at the PPKTP crystal per 404 nm laser pump pulse, n_p	0.0458	0.0214	0.0103
Measured herald (idler) photon rate R_h (counts/s)	9.73×10^5	4.75×10^5	2.32×10^5
Measured Detector 1 (herald channel) dark counts rate D_h (counts/s)	471	471	471
Calculated fractional contribution of dark counts to total herald counts D_h/R_h	4.84×10^{-4}	9.92×10^{-4}	2.03×10^{-3}
Measured heralded fluorescent rate R_{hf} (counts/s)	121	57.7	27.9
Measured Detector 2 (fluorescence channel) dark counts rate D_{f2} (counts/s)	30	30	30
Calculated fractional contribution from dark counts to total heralded fluorescent counts with 10 ns gate window $D_{f2} * R_h * [10 \text{ ns}]/R_{hf}$	2.41×10^{-3}	2.47×10^{-3}	2.49×10^{-3}
Calculated efficiency of heralded fluorescence $e_{hf} = R_{hf}/R_h$	1.244×10^{-4}	1.215×10^{-4}	1.204×10^{-4}
Calculated average time interval between successive heralded fluorescent counts $1/R_{hf}$ (ms)	8.26	17.3	35.9

Supplementary Table S2: **Heralded fluorescent photons from heralded single-photon excitation at three different average photon incident rates.**

Incident rates	Rate 1	Rate 2	Rate 3
Integration time for measuring coincidence between herald and fluorescent photons (s)	200	200	200
Measured peak counts of coincidence between herald and fluorescent photons	1,580	786	391
Integration time for measuring coincidence between herald and residual incident photons (instrument response function) (s)	100	-	-
Measured peak counts of coincidence between herald and residual incident photons (instrument response)	1,154	-	-
Extracted fluorescent lifetime (±95% confidence interval) (ns)	1.20 ± 0.05	1.21 ± 0.03	1.24 ± 0.03

Supplementary Table S3: **Experimental parameters and measured data for cross correlation between herald and fluorescent photons used to generate the plots in Fig. 1c of the main text. The bin size was 128 ps.**

V. THE SECOND ORDER COHERENCE FUNCTION AT ZERO TIME DELAY $g^{(2)}(t = 0)$ OF THE HERALDED INCIDENT PHOTONS AND THE HERALDED FLUORESCENT PHOTONS

A. Measured values of $g^{(2)}(t = 0)$ of heralded incident photons

To measure the second order coherence function at zero time delay $g^{(2)}(t = 0)$ of the heralded incident single photons, the Detector 2 (D2) in panel **a** of Fig. S2 is replaced by a 50:50 fiber beam splitter, followed by Detector 2 (D2) and Detector 3 (D3) on each output port. The count data needed to calculate $g^{(2)}(t = 0)$ are then recorded for each incident rate. The measured count data for a 10 s integration time and 6 ns coincidence/gate window are shown in Fig. S4 for three different incident photon rates, represented by the average number of photon pairs per pump pulse.

As described in the main text and in Ref. [10], the heralded (equivalently in this case, the conditional) value of $g^{(2)}(t = 0)$ can be calculated from

$$g^{(2)}(t = 0) = \frac{N_H \times N_C}{N_2 \times N_3}. \tag{1}$$

Here N_H is the herald count, N_C is the coincidence count between Detector 2 and Detector 3 when both are gated by herald detection, and N_2 (N_3) is the count at Detector 2 (Detector 3) when also gated by herald detection, i.e., by a count on Detector 1 (D1). The resulting $g^{(2)}(t = 0)$ values are reported in Fig. 2c of the main text.

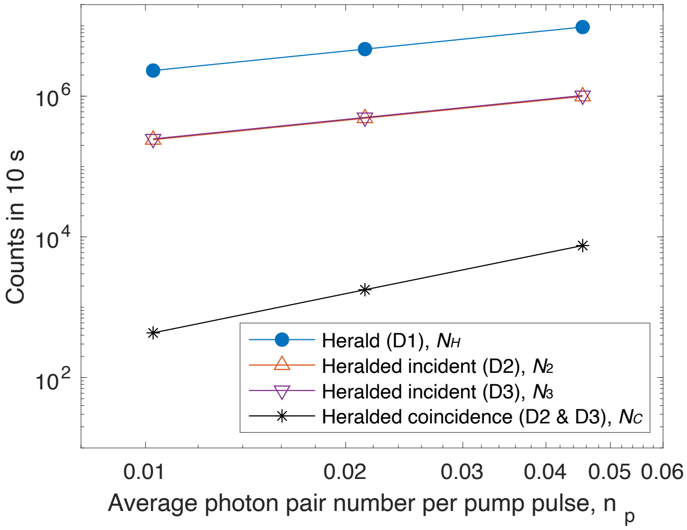

Supplementary Figure S4: Count data used to calculate the conditioned second order coherence function at zero time delay $g^{(2)}(t = 0)$ of the heralded incident single photons in Fig. 2c of the main text.

B. Measured value of $g^{(2)}(t = 0)$ of heralded fluorescent photons

The second order coherence function at zero time delay $g^{(2)}(t = 0)$ of the fluorescent photons emitted by the sample, which are also conditional on the detection of a herald photon in Detector 1 (D1), is similarly calculated from

$$g^{(2)}(t = 0) = \frac{N_H \times N_C}{N_2 \times N_3}$$

when Detectors 2 and 3 are located in the fluorescence module as indicated in Fig. 2a of the main text and Fig. S1. This is only measured for the highest incident rate (Rate 1). The measured counts are reported in the caption of Fig. 2 of the main text and the resulting $g^{(2)}(t = 0)$ value is reported in Fig. 2c and corresponding caption in the main text.

C. Theoretical estimation of $g^{(2)}(t = 0)$

We provide here a simple model to derive a theoretical estimate of the $g^{(2)}(t = 0)$ value for the heralded incident and fluorescent photons.

The output of a type II SPDC is a degenerate two-mode squeezed vacuum state and tracing over one of the modes leads to a thermal photon number distribution for the other mode, i.e., in a single arm of the SPDC, which is given by

$$P(n_p, n) = \frac{n_p^n}{(1 + n_p)^{n+1}}. \tag{2}$$

Here $n_p > 0$ is the average number of photon pairs per pump pulse, which is equal to the average photon number in one of the two arms, per pump pulse, and $n = 0, 1, 2, 3...$ is the number of photons in the state.

The herald detection then removes the zero-photon component of the above distribution and projects the incident beam onto the following distribution:

$$\begin{aligned} &P_h(n_p, n) = 0, \text{ for } n = 0, \\ &P_h(n_p, n) = \frac{1 + n_p}{n_p} \frac{n_p^n}{(1 + n_p)^{n+1}}, \text{ for } n = 1, 2, 3... \end{aligned} \tag{3}$$

With this projection the heralded incident beam has an average photon number of

$$\bar{n} = \sum_{n=1}^{+\infty} n \times P_h(n_p, n) = 1 + n_p. \tag{4}$$

Inserting $P_h(n_p, n)$ into the general expression

$$g^{(2)}(t = 0) = \frac{\langle n(n - 1)\rangle}{\langle n\rangle^2}, \tag{5}$$

where $\langle \ \rangle$ denotes averaging over the distribution, we obtain the value

$$g^{(2)}(t = 0) = \frac{2\, n_p}{1 + n_p}, \tag{6}$$

without any free parameters. In this simple model, the heralded fluorescent photons follows the same expression of $g^{(2)}(t = 0)$. This is plotted in Fig. 2c of the main text as the "Theory" curve, which agrees well with the measured values.

VI. DERIVATION OF PROBABILITY DISTRIBUTION OF THE NUMBER OF HERALDS PER FLUORESCENT PHOTON

A. Counting Scheme

The data and counting schematic of Fig. 3a of the main text applies to all the experimental and simulated probability distributions of the number of heralds per fluorescent photon. As we have described in the main text and as is quantified by the value of the $g^{(2)}(t=0)$ measurements, there is a small but finite probability of two photons in the heralded incident single-photon pulse, which then leads to a small probability of two fluorescent photons emitted from the sample. When the fluorescent counts are combined from the two detectors, such rare two-photon events in the fluorescence can also be present in the probability distribution of the number of heralds per fluorescent photon, depending on how we define the counting rule.

In this work we employ a counting rule that explicitly counts the number of herald photons observed between successively heralded fluorescent counts. Fig. S5 shows how a rare two-photon event in the fluorescence comes about and is counted as a contribution to $P(0)$ when this rule is followed. When there are two detected fluorescent photons (each from one detector) within a herald gate window, the first fluorescent photon will yield $N=1$ herald, while the second fluorescent photon will yield $N=0$ heralds, since obviously there is no herald detected between these two photons.

Because the dead time of detection (86 ns) is longer than the gate window (10 ns), any such pair of two detected photons within a gate window must derive from each photon coming from a different detector. Thus, it is easy to recognize that these rare two-photon events are exactly the same events measured in the coincidence between the two fluorescent channels gated by a herald detection, which is represented by the N_C counts employed in the calculation of the heralded $g^{(2)}(t=0)$ values for fluorescence (Section V). As reported in the main text, there are only 8 such events in 5 hours of measurements that yielded 1,668,407 heralded fluorescent detections, resulting in a $P(0)$ value of $\sim 4.8\times10^{-6}$.

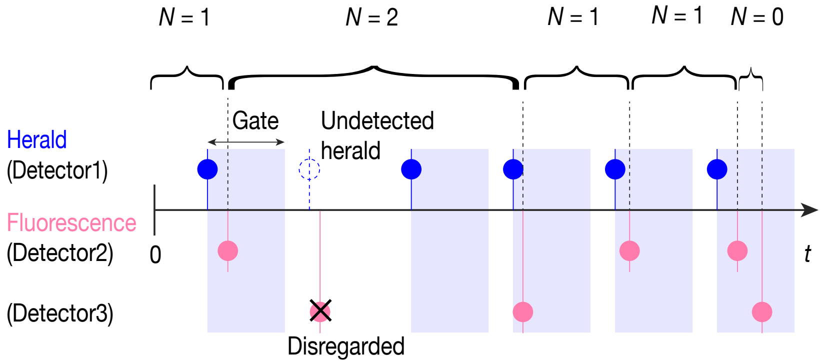

Supplementary Figure S5: Data schematic showing the counting scheme used to generate the probability distribution of the number of heralds N per fluorescent photon, with explicit examples for contributions to $P(N), N=0,1,2$.

B. Derivation of Eq. (1) in the main text and its asymptotic form

The statistical model underlying Eq. (1) of the main text establishes the distribution of $P(N)$, the probability of N heralded incident photons being detected between two consecutive detections of heralded fluorescent photons, where the latter are detected within the 10 ns gate window (Sec. IV and Fig. S5). Both incident and fluorescent photon detection events are conditioned on the detection of a herald photon, so fluorescent detections not associated with a herald detection are disregarded (see Fig. S5 above). The heralding also ensures that the efficiency of the herald channel detection does not appear in the stochastic model. The distribution $P(N)$ is then parameterized only by the probability of detection of a heralded fluorescent photon, which we equate with the efficiency of the heralded fluorescence, e_{hf}. All fluorescence detection events are treated as independent.

According to the counting scheme shown in Fig. S5, there can be no contribution from single incident photons to $P(0)$, since a non-zero incident photon contribution conditioned on detection of a herald photon can only occur if

there is more than one fluorescent photon emitted from the sample within the fixed time window associated with the herald detection and this is only possible if the incident photon pulse contained multiple photons. Here the statistical model assumes that the incident photon pulse contains only single photons, so the theoretical value of $P(0)$ is strictly zero. The accuracy of this assumption can be verified by the experimental measurement of $P(0)$ described above, which yielded the very small value $\sim 4.8 \times 10^{-6}$.

Under this assumption, for $N = 1$ the probability $P(1)$ is given by e_{hf}, the heralded fluorescence channel efficiency. For $N = 2$, we have $P(2) = (1 - e_{hf})e_{hf}$, which accounts for one herald count without an associated fluorescence within its gate time and a second herald count that is followed by an associated fluorescence within its gate time. For general $N \geq 1$ we then have

$$P(N) = (1 - e_{hf})^{N-1} e_{hf}, \tag{7}$$

which derives from a series of $N - 1$ consecutive herald counts each without an associated fluorescence, followed by a single herald count that is followed by an associated fluorescence within its gate time. This is the form of Eq. (1a) in the main text.

To obtain the asymptotic form in the limit $e_{hf} \ll 1$, we make use of the expansion $\ln(1 - \epsilon) = -\epsilon - O(\epsilon^2)$ for small ϵ, to obtain

$$\ln P(N) = (N - 1)\ln(1 - e_{hf}) + \ln e_{hf} \simeq -(N - 1)e_{hf} + \ln e_{hf} \tag{8}$$

from which it follows that $P(N) \propto \exp(-N e_{hf})$.

We note that with the detection conditioned on the presence of herald photons, there is no dependence on the dead time of the herald detector. Also, since the time scale between heralded fluorescent detections is significantly larger than the dead time of the fluorescent channel ($1/R_{hf} \geq 8.26$ ms, while $T_D = 86$ ns), there is no effect of the dead time on the heralded fluorescent detections.

C. Theoretical estimation of $P(0)$ considering multi-photon events in the heralded incident photons

Given the photon number distribution of the heralded incident single photon shown in Eq. (3) and following the counting scheme shown in Fig. S5, it is straightforward to estimate the value of $P(0)$ as

$$P(0) \sim \frac{[P_h(n_p, n = 2) * (1/2) * e_{hf} * e_{hf}] + [P_h(n_p, n = 3) * (6/8) * e_{hf} * (2 * e_{hf} - 2 * e_{hf}^2)]}{e_{hf}}. \tag{9}$$

Here for simplicity we have considered only the 2-photon and 3-photon components and neglected other higher order contributions. Inserting the measured values $n_p = 0.0458$ and $e_{hf} = 0.8 \times 1.244 \times 10^{-4}$ (the factor of 0.8 here is for the transmission of the additional fibre beam splitter added for the data shown in Fig. 3b) into Eq. (9), we obtain a value $P(0) \sim 2.4 \times 10^{-6}$, in good agreement with the experimental value of $(4.8 \pm 1.7) \times 10^{-6}$, with a factor of ~ 2.

VII. NUMERICAL SIMULATIONS OF THE PROBABILITY DISTRIBUTION OF THE NUMBER OF HERALDS PER FLUORESCENT PHOTON

A. Numerical simulation methodology

The SPDC produces correlated photon pairs, such that the detection of a photon in the herald channel will correlate with a photon incident upon the sample. Under our heralded (i.e., gated) detection scheme, every fluorescent photon is detected with conditional efficiency $e_{hf} = R_{hf}/R_h$, while the herald channel has a detection efficiency of 100% by definition. Thus two independent Bernoulli random variables with success probabilities 100% and e_{hf} summarize the ultimate success or failure to measure the herald and the heralded fluorescent photon, respectively.

Note that in the analysis of the previous section, no reference was made to the actual time duration of each laser pulse bin. All we required was that each pulse, or 'trial' is described by a pair of independent and identically distributed random variables. Therefore, the logic of the derivation is completely unchanged if we replace each pulse with its known periodic duration with the randomly generated pair of heralded fluorescent photon detection and heralded detection of an incident single photon. A numerical simulation that samples from sequences of pairs of independent Bernoulli trials can then faithfully sample from trajectories where a given number of heralds are separated by fluorescent photons. Only the exact timing information is lost in this simulation, which is irrelevant to the count of the number of heralds between fluorescent photons.

The numerical simulations were carried out by randomly sampling the herald and heralded fluorescent photon detection events according to the efficiencies of the respective channels, 100% and e_{hf}. The distribution of herald photon number per fluorescent photon detection was obtained by counting the number of herald photons recorded between successive heralded fluorescent records, in the same way as was done in the processing of the experimental data (Section VI).

B. Comparison of experimental distributions with simulated distributions for same data integration times

Incident rates	Rate 1	Rate 2	Rate 3
Calculated average number of photon pairs produced at the PPKTP crystal per 404 nm laser pump pulse, n_p	0.0458	0.0214	0.0103
Integration time (seconds)	4,000	4,000	4,000
Measured total herald counts	3,892,670,053	1,899,658,196	926,279,340
Measured total heralded fluorescent counts	484,427	230,783	111,560
Calculated raw $1/e_{hf} = R_h/R_{hf}$	8,036	8,231	8,303
Fitted N_0 of experimental distributions (±95% confidence interval)	$8,026 - 13/+19$	$8,203 - 27/+20$	$8,292 - 41/+41$
Fitted N_0 of simulated distributions (±95% confidence interval)	$8,026 - 19/+19$	$8,285 - 27/+28$	$8,292 - 41/+41$

Supplementary Table S4: **Summaries of parameters and data in Fig. S6**

The measured and derived parameters characterizing the experimental distribution of the number of heralds per fluorescent photon in Fig. S6 are summarized in Table S6. The parameter N_0 is the fitting parameter in the exponential form $P(N) \propto \exp(-N/N_0)$ used to fit the experimental and simulated data in Fig. S6. The analysis in Section VI above shows that N_0 should be equal to $1/e_{hf}$ when $e_{hf} \ll 1$. The experimental data in Table S6 show that this equality is very well satisfied. We note that the e_{hf} for the experimental data here is slightly larger than the corresponding value for the experimental data shown in Fig. 3b of the main text, for which there was an additional fiber beam splitter in the setup that introduced additional loss (with transmission ~ 0.8) in the fluorescent paths.

Fig. S6 shows a comparison of the experimental and the numerically simulated distribution of the number of heralds

per fluorescent photon for each of the three average incident rates shown in Fig. 2c the main text, each with integration time 4,000 s.

Panels **a-c** show the experimental distributions together with both the predictions of the analytical model of Eq. (1) (solid lines) and the numerical fit to the single exponential $P(N) = A_0 \exp(-N/N_0)$ (dashed lines). Each panel shows the results for one of the three average incident rates. The resulting fitted values for N_0 are $8,026 - 13/+19$, $8,203 - 27/+20$ and $8,292 - 41/+41$, within a 95% confidence interval. These values agree well with the experimental values $1/e_{hf}$, namely 8,036 and 8,231 and 8,303 (Table S4).

Panels **d-f** show the numerically simulated distributions, also together with the predictions of the analytical model of Eq. (1) (solid lines) and the numerical fit to the single exponential $P(N) = A_0 \exp(-N/N_0)$ (dashed lines). The fitted values of N_0 for these simulated distributions are $8,026 - 19/+19$, $8,285 - 27/+28$ and $8,292 - 41/+41$, with 95% confidence interval. These fits are also in good agreement with the experimental values of $1/e_{hf} = 8,036$ and 8,231 and 8,303, respectively (Table S4).

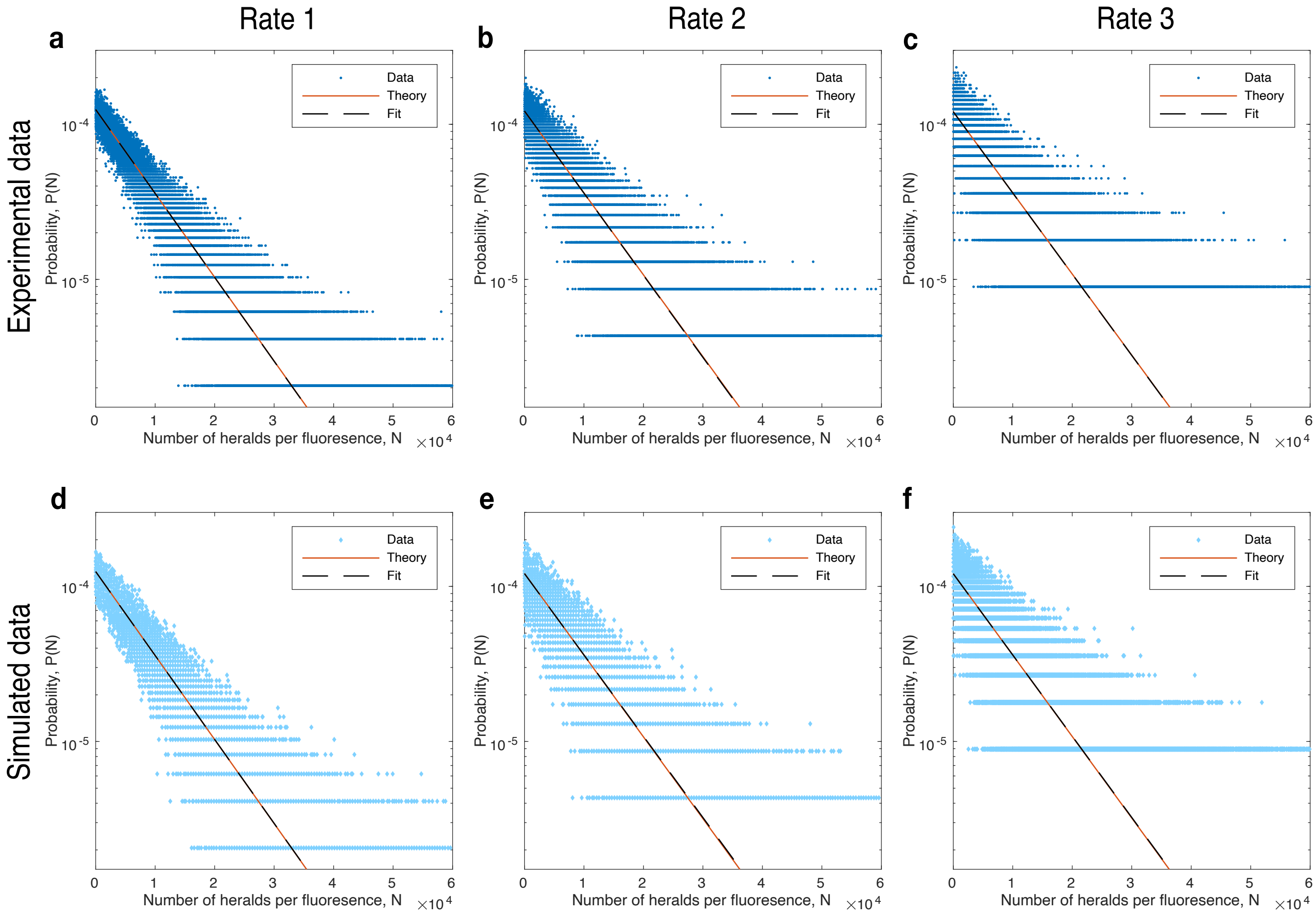

Supplementary Figure S6: **Fitting of the experimental and numerically simulated distributions according to $\mathbf{P(N) \propto \exp(-N/N_0)}$.**

C. Distribution of the simulated counts in the $N = 1$ bin

Fig. S7 shows the normalized distribution of the simulated counts in the $N = 1$ bin for 1000 simulations of 1800 s integration time each. The calculated mean is 15.6150 and the calculated standard deviation is 4.1172. The red circles and lines show a Poisson distribution with the same mean of 15.6150. This confirms that the distribution of the counts in the $N = 1$ bin follows a Poisson distribution, as expected from the independence between the individual events that are associated with a count in the bin.

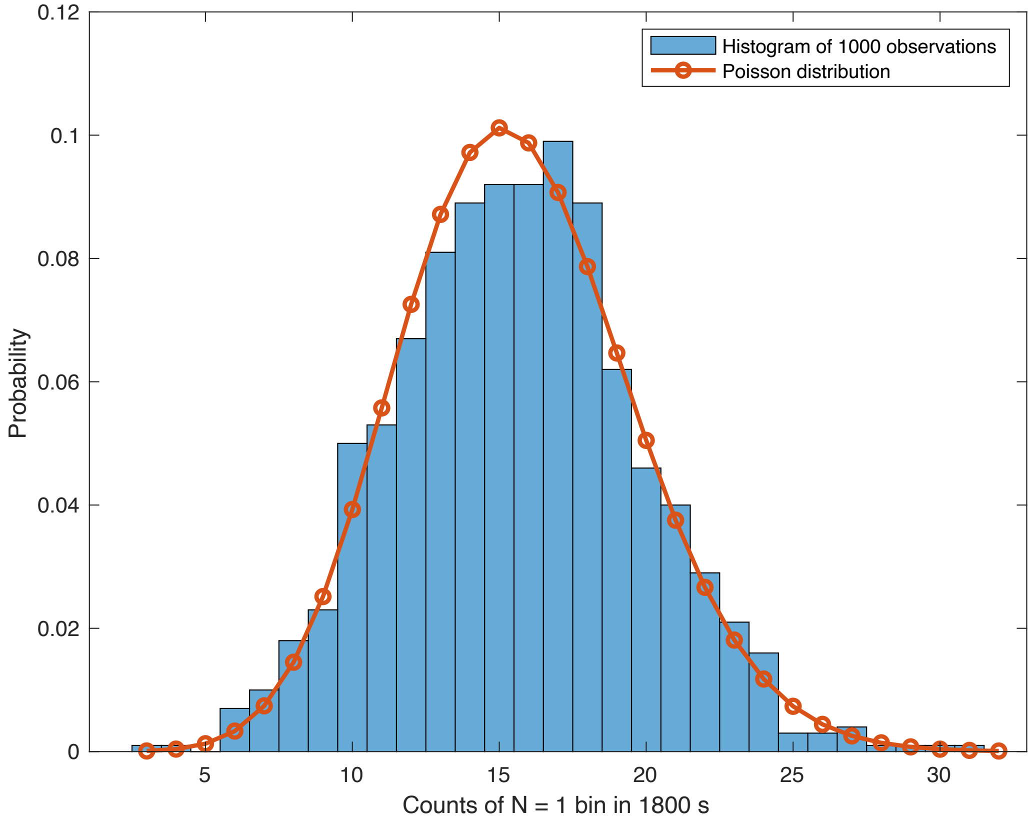

Supplementary Figure S7: **Distribution of the simulated counts at N = 1 bin confirming a Poisson distribution.**

D. Longer time numerical simulations of the probability distribution of the number of heralds per fluorescent photon

Fig. S8 shows the reduction of Poisson counting noise when running numerical Monte Carlo simulations for 1.8×10^6 s integration time (100 times the length of the experimental and simulated data in main Fig. 3b and Fig. 3c). For such large numbers of event counts (in total 1,777,364,962,000 heralds and 166,855,955 heralded fluorescent photons), the Poisson counting noise is sufficiently reduced. The light blue diamonds (which are heavily overlapping in the plots), are the simulated distribution obtained with the experimental efficiency e_{hf}. The red solid curve is derived from the analytical stochastic model of Eq. (1) of the main text using the efficiency e_{hf}. (See also Fig. 3b and Fig. 3c of the main text). The inset zooms into the bins at $N = 1, ...50$, showing the data points in each bin together with error bars representing Poisson standard deviations ($\sqrt{\text{counts at } N \text{ bin}}$ normalized by total counts of all bins).

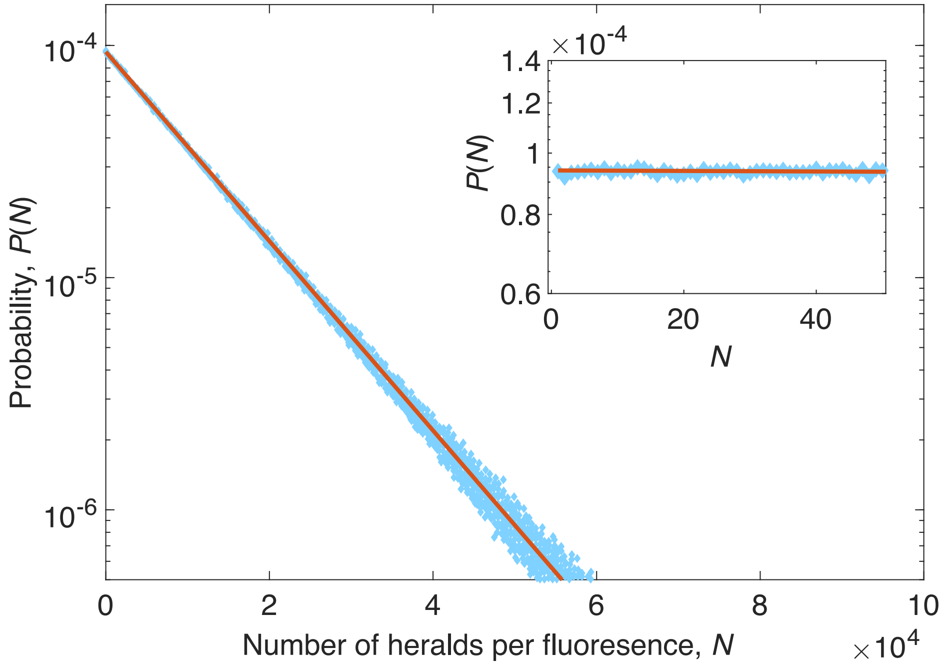

Supplementary Figure S8: Numerical Monte Carlo simulation with an extremely large number of event counts (in total 1,777,364,962,000 heralds and 166,855,955 heralded fluorescent photons) showing the reduction in noise obtained by running for longer times.

[1] Sabines-Chesterking, J. *et al.* Sub-shot-noise transmission measurement enabled by active feed-forward of heralded single photons. *Phys. Rev. Applied* **8**, 014016 (2017).

[2] Tong, A. L. *et al.* Comparison of the energy-transfer rates in structural and spectral variants of the b800–850 complex from purple bacteria. *The Journal of Physical Chemistry B* **124**, 1460–1469 (2020).

[3] Ogren, J. I. *et al.* Impact of the lipid bilayer on energy transfer kinetics in the photosynthetic protein lh2. *Chem. Sci.* **9**, 3095–3104 (2018).

[4] Blankenship, R. E. *Molecular Mechanisms of Photosynthesis, 2nd Edition* (Wiley-Blackwell, Chichester, West Sussex, 2014), 2 edition edn.

[5] Chan, H. C., Gamel, O. E., Fleming, G. R. & Whaley, K. B. Single-photon absorption by single photosynthetic light-harvesting complexes. *Journal of Physics B: Atomic, Molecular and Optical Physics* **51**, 054002 (2018).

[6] Monshouwer, R., Abrahamsson, M., Van Mourik, F. & Van Grondelle, R. Superradiance and exciton delocalization in bacterial photosynthetic light-harvesting systems. *The Journal of Physical Chemistry B* **101**, 7241–7248 (1997).

[7] Schlau-Cohen, G. S., Wang, Q., Southall, J., Cogdell, R. J. & Moerner, W. Single-molecule spectroscopy reveals photosynthetic lh2 complexes switch between emissive states. *Proceedings of the National Academy of Sciences* **110**, 10899–10903 (2013).

[8] Polívka, T., Pullerits, T., Frank, H. A., Cogdell, R. J. & Sundström, V. Ultrafast formation of a carotenoid radical in lh2 antenna complexes of purple bacteria. *The Journal of Physical Chemistry B* **108**, 15398–15407 (2004).

[9] Monger, T. G., Cogdell, R. J. & Parson, W. W. Triplet states of bacteriochlorophyll and carotenoids in chromatophores of photosynthetic bacteria. *Biochimica et Biophysica Acta (BBA)-Bioenergetics* **449**, 136–153 (1976).

[10] Beck, M. Comparing measurements of g (2)(0) performed with different coincidence detection techniques. *JOSA B* **24**, 2972–2978 (2007).
